# Adaptive shut-down of EEG activity predicts critical acidemia in the near-term ovine fetus

**DOI:** 10.14814/phy2.12435

**Published:** 2015-07-06

**Authors:** Martin G Frasch, Lucien Daniel Durosier, Nathan Gold, Mingju Cao, Brad Matushewski, Lynn Keenliside, Yoram Louzoun, Michael G Ross, Bryan S Richardson

**Affiliations:** 1 Department of Obstetrics and Gynaecology, Department of Neurosciences, CHU Ste-Justine Research Center, Université de MontréalMontreal, Quebec, Canada; 2 Department of Mathematics and Statistics, York UniversityToronto, Ontario, Canada; 3 Department of Obstetrics and Gynecology, University Western OntarioLondon, Ontario, Canada; 4 Imaging Program, Lawson Health Research InstituteLondon, Ontario, Canada; 5 Department of Mathematics, Bar-Ilan UniversityRamat-Gan, Israel; 6 Department of Obstetrics & Gynecology, LA BioMed at Harbor-UCLA Medical CenterTorrance, California

**Keywords:** Acidosis, asphyxia, ECOG, EEG, Fetus, FHR, hypoxia, monitoring

## Abstract

In fetal sheep, the electrocorticogram (ECOG) recorded directly from the cortex during repetitive heart rate (FHR) decelerations induced by umbilical cord occlusions (UCO) predictably correlates with worsening hypoxic-acidemia. In human fetal monitoring during labor, the equivalent electroencephalogram (EEG) can be recorded noninvasively from the scalp. We tested the hypothesis that combined fetal EEG – FHR monitoring allows for early detection of worsening hypoxic-acidemia similar to that shown for ECOG-FHR monitoring. Near-term fetal sheep (*n* = 9) were chronically instrumented with arterial and venous catheters, ECG, ECOG, and EEG electrodes and umbilical cord occluder, followed by 4 days of recovery. Repetitive UCOs of 1 min duration and increasing strength (with regard to the degree of reduction in umbilical blood flow) were induced each 2.5 min until pH dropped to <7.00. Repetitive UCOs led to marked acidosis (arterial pH 7.35 ± 0.01 to 7.00 ± 0.03). At pH of 7.22 ± 0.03 (range 7.32–7.07), and 45 ± 9 min (range 1 h 33 min–20 min) prior to attaining pH < 7.00, both ECOG and EEG amplitudes began to decrease ∼fourfold during each FHR deceleration in a synchronized manner. Confirming our hypothesis, these findings support fetal EEG as a useful adjunct to FHR monitoring during human labor for early detection of incipient fetal acidemia.

## Introduction

Human clinical studies indicate an increasing risk for neonatal adverse outcomes and longer term sequellae (e.g., cerebral palsy) with umbilical cord pH values <7.00 (Liston et al. 2002). This is supported by studies in the ovine fetus showing that pre-existing hypoxia alters cerebral and cardiovascular responses to labor-like umbilical cord occlusions (UCOs) (Gardner et al. 2002a; Fletcher et al. 2006; Wassink et al. 2013). This has led to the use of electronic fetal heart rate (FHR) monitoring as the mainstay for assessing fetal health during labor (Liston et al. 2002). The absence of FHR decelerations along with the presence of FHR variability is highly predictive for normal fetal blood gas/pH at birth (Liston et al. 2002). However, clinical FHR monitoring has a low positive predictive value (PPV) for clinically important acidemia at birth (PPV ∼50%) (Liston et al. 2002). Consequently, there is continued need for improving existing or introducing new technologies for the detection of fetal hypoxic-acidemia during labor.

We recently studied patterns of electrocortical activity (ECOG) recorded from the cortex as well as FHR in the near-term ovine fetus in response to repetitive UCOs insults as might be seen in human labor. Our goal was to delineate the time-course and correlation of ECOG change with worsening acidemia (Frasch et al. 2011). There were consistent changes in ECOG with amplitude suppression and frequency increase during FHR decelerations accompanied by pathological decreases in fetal arterial blood pressure (ABP). These changes in ECOG suggested an “adaptive brain shutdown” and occurred on average 50 min prior to attaining a severe degree of acidemia (defined as fetal arterial pH < 7.00). Our findings suggested that fetal EEG monitoring during labor can improve early detection of acidemia.

As a first step toward implementing this technology in human labor surveillance of fetal well-being we have shown that fetal electroencephalogram (EEG), the clinically available equivalent of the ECOG, can be acquired with a modified FHR scalp electrode similar to that used during human labor (Frasch et al. 2010, 2012). Accordingly, this ancillary surveillance modality during labor could be added easily and cost-effectively to the current electronic FHR monitoring that is widely used (Liston et al. 2002). We hypothesized that fetal EEG recorded from a modified FHR scalp electrode will allow for early detection of worsening acidemia similar to our previous findings for fetal ECOG (Prout et al. 2010; Frasch et al. 2011). Consequently, in this study we subjected near-term ovine fetuses to repetitive UCO insults and compared the fetal ECOG and EEG responses during worsening acidemia. Our findings validate the original observations made in a different cohort with a different UCO paradigm (Frasch et al. 2011). Moreover, we demonstrate that early detection of acidemia is indeed possible from scalp EEG recorded using modified FHR electrode.

## Materials and Methods

### Surgical preparation

Nine near-term ovine fetuses (123 ± 2 days gestational age (GA), term = 145 days) of mixed breed were surgically instrumented. The anesthetic and surgical procedures and postoperative care of the animals have been previously described (Kaneko et al. 2003; Frasch et al. 2009). Briefly, polyvinyl catheters were placed in the right and left brachiocephalic arteries and the right cephalic vein. Stainless steel electrodes were sewn onto the fetal chest to monitor the electrocardiogram (ECG). Stainless steel electrodes were additionally implanted biparietally on the dura for the recording of ECOG. A modified double spiral FHR electrode was placed midline just anterior to the ECOG electrodes to acquire the EEG (Frasch et al. 2010, 2012). An inflatable silicon rubber cuff (In Vivo Metric, Healdsburg, CA) for UCO induction was also placed around the proximal portion of the umbilical cord and secured to the abdominal skin. Once the fetus was returned to the uterus, a catheter was placed in the amniotic fluid cavity and another in the maternal femoral vein. Antibiotics were administered intravenously to the mother (0.2 g trimethoprim and 1.2 g sulfadoxine, Schering Canada Inc., Pointe-Claire, Canada) and the fetus and into the amniotic cavity (1 million IU penicillin G sodium, Pharmaceutical Partners of Canada, Richmond Hill, Canada). Amniotic fluid lost during surgery was replaced with warm saline. The uterus and abdominal wall incisions were sutured in layers and the catheters exteriorized through the maternal flank and secured to the back of the ewe in a plastic pouch.

Postoperatively, animals were allowed 4 days to recover prior to experimentation and daily antibiotic administration was continued. Arterial blood was sampled for evaluation of fetal condition and catheters were flushed with heparinized saline to maintain patency. Animals were 129 ± 1 days GA on the first day of experiments. Animal care followed the guidelines of the Canadian Council on Animal Care and was approved by the University of Western Ontario Council on Animal Care.

## Experimental procedure

The animals were studied over a ∼6 h period in two groups. After a 1–2 h baseline control period, both groups of animals underwent mild, moderate, and severe series of repetitive UCOs by graduated inflation of the occluder cuff with a saline solution. During the first hour following the baseline period, mild variable FHR decelerations were induced with a partial UCO for 1 min duration every 2.5 min, with the goal of decreasing fetal heart rate by ∼30 bpm, corresponding to an ∼50% reduction in umbilical blood flow (Itskovitz et al. 1983; Richardson et al. 1989). During the second hour, moderate variable FHR decelerations were induced with stronger partial UCOs for 1 min duration every 2.5 min with the goal of decreasing fetal heart rate by ∼60 bpm, corresponding to an ∼75% reduction in umbilical blood flow (Itskovitz et al. 1983; Richardson et al. 1989). Animals then developed severe variable FHR decelerations provoked by complete UCO for 1 min duration every 2.5 min until the targeted fetal arterial pH of less than 7.0 was detected or 2 h of severe UCO had been carried out, at which point the repetitive UCOs were terminated. All animals were then allowed to recover for 48 h following the last UCO. Fetal arterial blood samples were drawn at baseline, at the end of the first UCO of each series (mild, moderate, severe), and at 20 min intervals (between UCOs) throughout each of the series, as well as at 1, 24, and 48 h of recovery. For each UCO series blood gas sample and the 24 h recovery sample, 0.7 mL of fetal blood was withdrawn, whereas 4 mL of fetal blood was withdrawn at baseline, at pH nadir less than 7.00, and at 1 h and 48 h of recovery. The amounts of blood withdrawn were documented for each fetus and replaced with an equivalent volume of maternal blood at the end of day 1 of study.

All blood samples were analyzed for blood gas values, pH, glucose, and lactate with an ABL-725 blood gas analyzer (Radiometer Medical, Copenhagen, Denmark) with temperature corrected to 39.0°C. Plasma from the 4 mL blood samples was frozen and stored for cytokine analysis, and will be reported separately.

After the 48-h recovery blood sample, the ewe and the fetus were killed by an overdose of barbiturate (30 mg sodium pentobarbital IV, MTC Pharmaceuticals, Cambridge, Canada). A postmortem was carried out during which fetal sex and weight were determined and the location and function of the umbilical occluder were confirmed. The fetal brain was perfusion fixed and subsequently dissected and processed for later immunohistochemical study (data reported separately) as previously reported (Keen et al. 2011).

### Data acquisition and analysis

A computerized data acquisition system was used to record fetal arterial and amniotic pressures, the ECG, ECOG, and EEG electrical signals, as previously described (Richardson and Gagnon 2008), which were monitored continuously throughout the baseline period, UCO series, and first hour of the recovery period. Arterial and amniotic pressures were measured using Statham pressure transducers (P23 ID; Gould Inc., Oxnard, CA). Arterial blood pressure (ABP) was determined as the difference between instantaneous values of arterial and amniotic pressures. A PowerLab system was used for data acquisition and analysis (Chart 7 For Windows, ADInstruments Pty Ltd, Castle Hill, Australia).

Pressures, ECG, ECOG, and EEG were recorded and digitized at 1000 Hz for further study. For ECG, a 60 Hz notch filter was applied, whereas for ECOG and EEG, a band pass 0.3–30 Hz filter was used. FHR was triggered and calculated online from arterial pressure systolic peaks.

Averaged values of FHR and ABP were calculated from artifact-free recordings of 1 h of baseline, as well as between and during each consecutive variable FHR deceleration induced by the mild, moderate, and severe UCOs as previously reported (Ross et al. 2013). FHR_nadir_ was measured as the minimal FHR during a UCO; ABP_max_ was measured as maximal ABP during a UCO; ABP_min_ was measured as the minimal ABP during a UCO; ΔFHR was calculated as FHR deceleration depth during UCO, that is, the difference between mean FHR between UCO and FHR_nadir_; ΔABP was calculated as the difference between ABP_max_ and mean ABP between UCO; ΔABP_UCO_ was calculated as the difference between ABP_max_ and ABP_min_ to capture the known biphasic change of ABP during each UCO.

The ECOG and EEG signals were sampled down to 100 Hz prior to the ECOG and EEG analysis. Subsequently, the voltage amplitude and 95% spectral edge frequency (SEF), this being the ECOG/EEG frequency below which 95% of ECOG/EEG spectral power is found, were calculated over 3 sec intervals for the duration of the experimental monitoring. For each animal, mean values of the ECOG and EEG amplitudes and SEF were determined at baseline as well as during and between UCO for each of the deceleration series. To track the correlation between the ECOG/EEG and FHR, we determined the cross-correlation function (CCF) between the smoothed ECOG/EEG amplitudes (absolute value of the ECOG/EEG signals) and the smoothed FHR with a square smoothing kernel of 10 sec with delays of −100 to 100 sec (Matlab, Mathworks, Natick, MA). CCF analysis tests the spectrographic similarity of ECOG and EEG recordings. The CCF was normalized to 1 and maxima of the normalized CCF was determined. The closer these maxima (CCFM) are to 1, the higher is the correlation between both signals. We then compared the correlation between the different levels of occlusion (i.e., baseline, mild, moderate, and severe UCO) as a function of the delay:

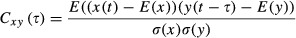
where *σ* (*x*) is its standard deviation, and *E*(*x*) is its expected value.

To further validate the degree of synchronization between ECOG/EEG amplitudes and FHR, we compared the coherence in the 0.01–0.1 Hz band – representing the 10–100 sec locking period – to all other frequency bands. A frequency range of 0.01–0.1 Hz represents the expected coherence time scale between these two signals. The spectral coherence is the ratio between the squared Fourier transform of the cross-correlation function divided by the Fourier transform of the correlation function of each signal by itself. As the correlated ECOG/EEG-FHR activities were consistently observed during the severe UCO series, the above comparisons were made between the severe UCO series versus all the preceding experimental stages (i.e., baseline, mild, and moderate UCO).

### Statistical analysis

Normal data distribution was tested using the Kolmogorov–Smirnov test followed by parametric or nonparametric tests, as appropriate. Arterial lactate, BE, glucose, and O_2_Sat measurements in response to repetitive UCOs and associated variable decelerations were compared with the corresponding baseline values by one-way repeated-measures analysis of variance analysis (ANOVA) or Friedman on ranks, adjusting for multiple comparisons with Holm–Sidak or Dunn’s method, for normally and non-normally distributed data, respectively. One-way repeated-measures ANOVA followed by Holm–Sidak (vs. baseline) or Student–Newman–Keuls (pairwise) tests for multiple comparisons have been used to assess differences in ECOG/EEG and cardiovascular responses to UCO. Differences between ECOG and EEG were assessed using the t-test or signed rank test. Differences in ECOG/EEG and cardiovascular alterations during ECOG/EEG-FHR synchronized pattern were tested using the t-test or signed rank test. A two-sided rank sum test was used to detect changes in CCF and spectral coherence between the ECOG/EEG amplitudes and FHR during the severe UCO series versus the preceding stages of the experiment (i.e., baseline, mild, and moderate UCO).

All values are expressed as means ± SEM. Statistical significance was assumed for *P* < 0.05. Pearson or Spearman correlation analysis was performed as appropriate, and *R* values are presented where *P* < 0.05 (SPSS 19; IBM, Armonk, NY).

## Results

### Fetal cardiovascular and brain response to umbilical cord occlusions

We measured cardiovascular (FHR and ABP) and brain electrical (ECOG/EEG) responses to UCO mimicking human labor in near-term ovine fetuses.

During baseline, fetal acid-base status, cardiovascular, and ECOG/EEG behavior were within normal physiologic range. Fetal arterial pH measured 7.35 ± 0.01, FHR was 159 ± 5 bpm and ABP was 44 ± 2 mmHg (Table1). Baseline EEG amplitude measured 37 ± 4 *μ*V, which was 3.5× lower than ECOG amplitude at 127 ± 14 *μ*V (*P* = 0.01, Table1). Baseline EEG SEF was 4.4 ± 0.3 Hz and 1.5× lower than baseline ECOG SEF at 6.7 ± 0.6 Hz (*P* < 0.01).

**Table 1:** Brain and cardiovascular responses to repetitive umbilical cord occlusions.

	Baseline	Mild UCO		Mod UCO		Sev UCO		Pattern	
		dur UCO	btw UCO	dur UCO	btw UCO	dur UCO	btw UCO	dur UCO	btw UCO
ECoG amplitude, *μ*V	127 ± 14	130 ± 14	131 ± 14	152 ± 29	152 ± 29	140 ± 24	156 ± 26	102 ± 17^2^	209 ± 26
ECoG SEF (95%), Hz	6.7 ± 0.6	8.2 ± 0.5^1^	8.1 ± 0.5^1^	7.6 ± 0.3	7.5 ± 0.4	6.0 ± 0.4	5.9 ± 0.3	11.0 ± 0.4^2^	8.6 ± 0.5
EEG amplitude, *μ*V	37 ± 4^4^	61 ± 13^4^	66 ± 14^4^	48 ± 11^4^	56 ± 12^4^	73 ± 11^4^	102 ± 13^1^	81 ± 8^2^	138 ± 17^4^
EEG SEF (95%), Hz	4.4 ± 0.3^4^	5.2 ± 0.9^4^	5.0 ± 0.9^4^	5.4 ± 0.8^4^	4.3 ± 0.4^4^	4.7 ± 0.4^4^	4.4 ± 0.2^4^	9.0 ± 0.7^2^	8.1 ± 0.7
FHR, bpm	159 ± 5	165 ± 5	162 ± 3	131 ± 6^1^	153 ± 2	115 ± 10^1^	162 ± 3	101 ± 6^2^	171 ± 8
ABP, mmHg	44 ± 2	48 ± 2^1^	49 ± 2^1^	54 ± 2^1^	53 ± 2^1^	60 ± 2^1^	60 ± 1^1^	57 ± 2	60 ± 2
ABP_max_, mmHg		64 ± 3^2^		74 ± 3^2^		88 ± 2^2^		65 ± 3	
ABP_min_, mmHg		32 ± 2^2^		37 ± 2		36 ± 2		33 ± 3	
FHR_nadir_, bpm		123 ± 8^2,3^		95 ± 5^2^		63 ± 5^2^		80 ± 6	
ΔFHR, bpm		39 ± 8^2,3^		59 ± 4^2,3^		99 ± 7^2^		90 ± 11	
ΔABP_UCO_, mmHg		33 ± 3		37 ± 3		52 ± 2^2,3^		32 ± 2	
ΔABP, mmHg		16 ± 2^2,3^		20 ± 2^2,3^		28 ± 2^2,3^		4 ± 3	

UCO, umbilical cord occlusion; during, during UCO; between, between UCO; ECoG, electrocorticogram; EEG, electroencephalogram; SEF, 95% spectral edge frequency of ECoG or EEG; Cardiovascular measures “between UCO”: FHR, fetal heart rate; ABP, mean arterial blood pressure; Cardiovascular measures “during UCO”: ABP_max_, maximal ABP during a UCO; ABP_min_, minimal ABP during a UCO; FHR_nadir_, minimal FHR during a UCO; ΔFHR, FHR deceleration depth during UCO; ΔABP_UCO_, difference between ABP_max_ and ABP_min_ during UCO; ΔABP, difference between ABP_max_ and mean ABP between UCO.

“Pattern” denotes the time segment of the UCO series when synchronized ECOG/EEG-FHR activities were observed (i.e., adaptive brain shut-down).

Mean ± SEM. ^1^, versus baseline; ^2^, pairwise (*i.e*., during vs. between UCO or vs. another “during UCO” measurement for ABP_max_, FHR_nadir_, ΔFHR, ΔABP, and ΔABP_UCO_); ^3^, versus the respective variable during Pattern; ^4^, EEG versus ECoG at the same time point.

**Figure 1:**
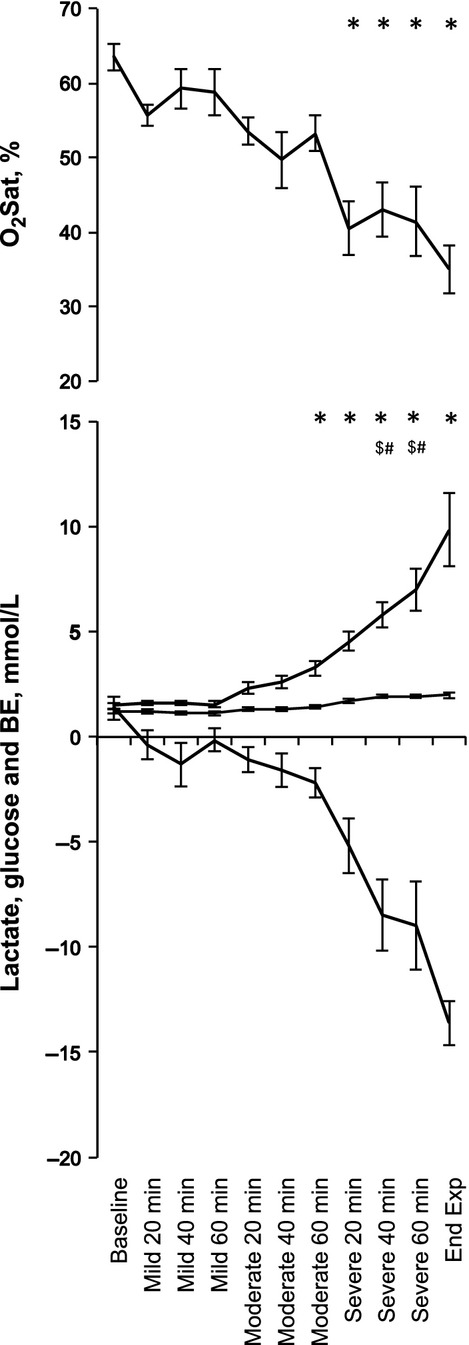
Acid-base status, arterial oxygen saturation, and glucose during the experiment’s baseline and umbilical cord occlusions (UCOs). Blood samples taken between the mild, moderate, and severe UCO series as well as at pH < 7.00 (End Exp). Changes in pH, pO2 and pCO2 have been presented elsewhere (Wang et al. 2014). *, base excess (BE) and O_2_Sat; $, glucose; #, Lactate – all vs. baseline.

As reported, repetitive UCO and associated variable FHR decelerations resulted in severe fetal acidosis (pH 7.35 ± 0.01 to 7.00 ± 0.03; BE 1.4 ± 0.6 to −13.6 ± 1.1 mEq/L) by the end of the severe UCO series (all *P* < 0.01, Fig.1). (Wang et al. 2014) We observed a steady decline in BE and O_2_Sat and increase in lactate and glucose, which were significant during the severe UCO series compared to the respective baseline values (Fig.1).

ABP increased on average to 75 ± 3 mmHg during each UCO versus 54 ± 2 mmHg between each UCO (*P* < 0.05). FHR deceleration depth averaged 66 ± 6 bpm (decreasing to 94 ± 6 during each UCO vs. 159 ± 3 bpm between each UCO, *P* < 0.05). Table1 provides a detailed account of the progressive FHR and ABP changes during each UCO series. Of note, ΔABP_UCO_ showed the expected dynamic behavior with increasing drop during each subsequent UCO series.

**Figure 2:**
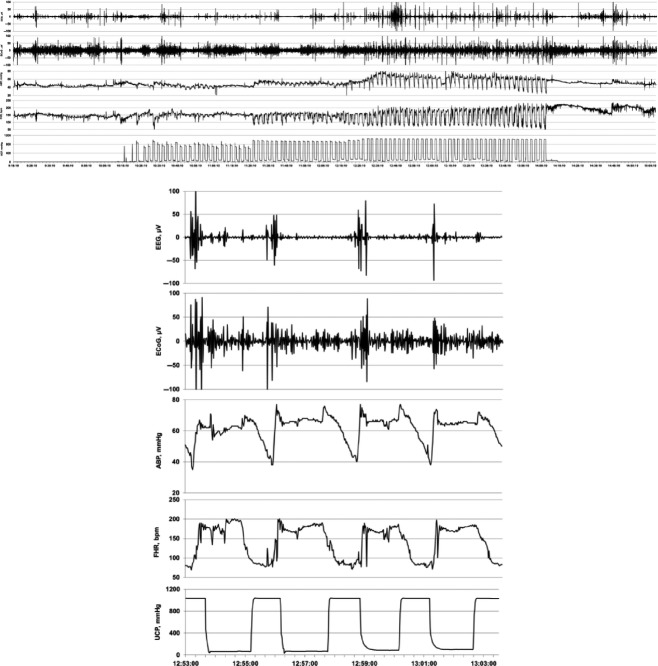
Example of an individual ECOG / EEG response to repetitive UCO. TOP: 60 min view of the adaptive brain shut-down pattern visible in ECOG and EEG in response to changes in arterial blood pressure (ABP) and fetal heart rate (FHR). BOTTOM: 10 min zoomed-in window of this pattern.

**Figure 3:**
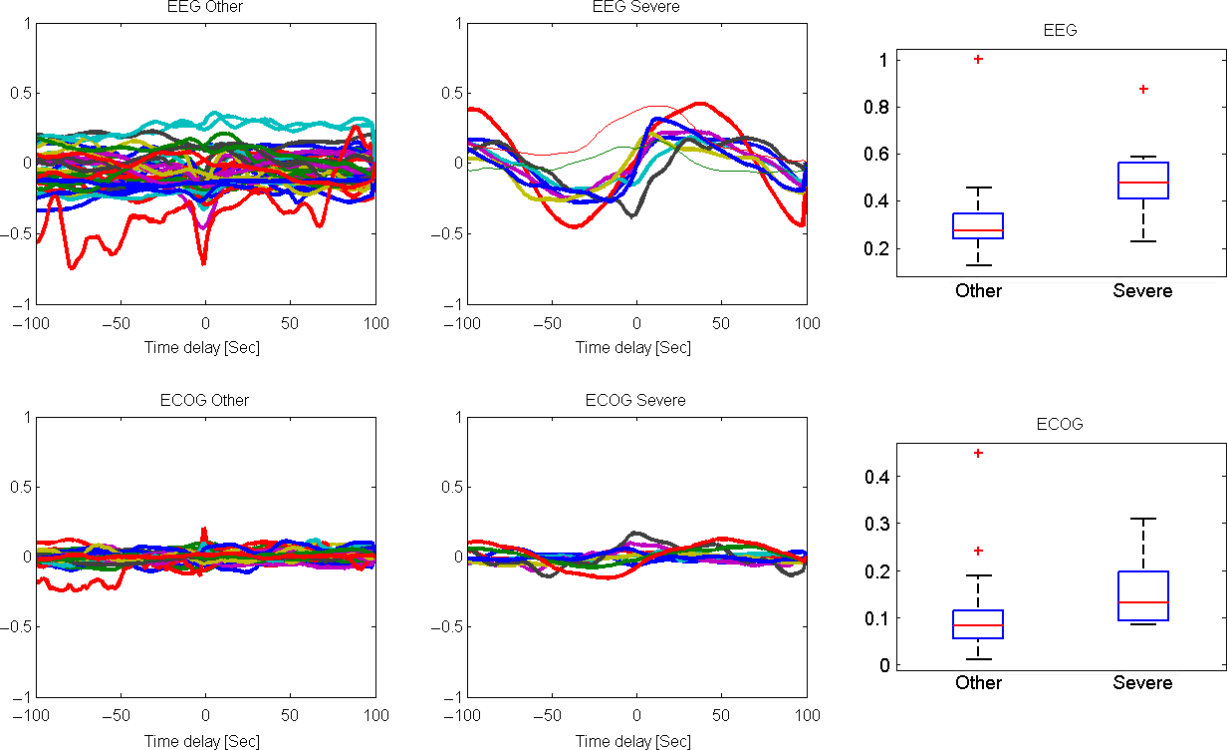
Cross-correlation function (CCF) analysis of the ECOG/EEG response to FHR decelerations. The baseline, mild, and moderate umbilical cord occlusion (UCO) series (denoted “other”) were compared with the ‘Severe’ UCO series when the adaptive brain shut-down was observed in all fetuses. The four leftmost plots represent the cross-correlation function between the smoothed ECOG/EEG amplitude and FHR with a 10 sec moving average with a delay ranging from −100 sec to 100 sec. For example, a time delay of 0 represents the correlation of the ECOG and FHR values at the same time. When a positive delay of 100 sec is used, the ECOG at time *t* is compared with the FHR at time *t* + 100. The two leftmost plots are the “other” groups, and the middle plot is the severe group when the adaptive brain shut-down was mostly observed in all fetuses. The upper plots are for the EEG/FHR correlation and the lower plots are for the ECOG/FHR. One can see that the amplitude of correlations is much higher in the severe group than in all other groups (*P* < 0.01 and *P* < 0.001, for EEG and ECOG, respectively), and that the amplitude is much larger in the EEG/FHR correlation than in the ECOG/FHR. The difference in the correlation amplitude is presented as a box plot in the two rightmost plots, with the red bar representing the median of the distribution and the box size represent the 25–75^th^ percentiles. ^+^ signifies the outliers of the distributions (beyond the 90^th^ percentile values). High amplitude of the correlation between EEG and FHR implies a strong synchronization between the two signals with an average delay of 30 sec.

Similar to earlier observations in a different cohort (Frasch et al. 2011), here we found that the ECOG began to show cyclical behavior correlated with UCO-induced FHR decelerations 48 ± 11 min (range 1 h 41 min–20 min) prior to the pH dropping <7.00. This corresponded to pH of 7.22 ± 0.03 (range 7.32–7.07) and O_2_Sat of 38 ± 2% (range 28–52%). During each UCO compared to the respective values between each UCO, ECOG SEF began to consistently increase to 11.0 ± 0.4 Hz from 8.6 ± 0.5 Hz (*P* < 0.01) and ECOG amplitude began to consistently decrease to 102 ± 17 *μ*V from 209 ± 26 *μ*V (*P* < 0.001) (Figs.2 and 3, Table1).

The EEG behaved similarly to the cyclical ECOG behavior and likewise correlated with UCO-induced FHR decelerations. The EEG began to show cyclical behavior correlated with UCO-induced FHR decelerations at a pH of 7.22 ± 0.03 (range 7.32–7.07), and 45 ± 9 min (range 1 h 33 min–20 min) prior to the pH dropping <7.00. During each UCO compared to the respective values between each UCO, EEG SEF began to consistently increase to 9.0 ± 0.7 Hz from 8.1 ± 0.7 Hz (*P* < 0.05) and amplitude began to consistently decrease to 81 ± 8 *μ*V from 138 ± 17 *μ*V (*P* < 0.001) (Figs.2 and 3).

ABP began to consistently show pathologically low increases of 4 ± 3 mmHg with each FHR deceleration 50 ± 14 min (range 2 h 06 min–19 min) prior to pH < 7.00 (Table1). This was in contrast to the ABP dynamics during the mild, moderate, and severe UCOs before the onset of this ABP behavior (*P* < 0.05, ΔABP, but not ΔABP_UCO_, Table1). The timing of the observed ECOG and EEG amplitude and SEF recurring pattern changes was highly correlated to the timing of the onset of the pathological ΔABP (both *R* = 0.99, *P* < 0.001) (Fig.2).

The onset timing of the ECOG/EEG-FHR synchronization and the respective O_2_Sat and pH values for individual animals showed a considerable spread. Hence, we tested if the onset timing correlated to the O_2_Sat or pH levels. We found a correlation at Pearson *R* = 0.86 (*P* = 0.003) and *R* = 0.76 (*P* = 0.02), that is, fetuses with an earlier onset of the ECOG/EEG-FHR synchronized pattern (i.e., farther in time from the pH nadir) showed higher O_2_Sat and pH values; inversely, the more hypoxic fetuses showed the pattern closer to the pH nadir.

### Evidence of ECOG/EEG – FHR synchronization

To test for the assumption that ECOG/EEG and FHR show temporal synchronization early and prior to the onset of severe acidemia (Frasch et al. 2011), we computed in each group the delayed correlation between the smoothed ECOG/EEG amplitudes and smoothed FHR with a 10 sec moving average with a delay ranging from −100 sec to 100 sec (Fig.3). If for example, the EEG and FHR are synchronized exist with a delay, we expect a positive correlation between the ECOG/EEG and FIHR amplitudes with the appropriate delay. If there is no synchrony, we expect the correlation to be near 0 for all delays.

Only during the severe UCO series did a clear correlation with a phase lag appear between ECOG/EEG amplitude and FHR. Of note, the result was more pronounced for EEG-FHR cross-correlation function (CCF) than for ECOG-FHR CCF.

Note that the correlation switches signs. Thus, for some delays, the signals are correlated and for others, they are anticorrelated. Such a difference emerges when two wave functions are compared with some delay. We checked the distribution of the difference between the maximal and minimal correlation as a function of the phase in all sheep fetuses. This difference was significantly larger during the severe UCO series compared to all previous time periods (baseline, mild, and moderate UCO series), with an average difference of 0.16 ± 0.08 (ECOG-FHR CCF) and 0.5 ± 0.16 (EEG-FHR CCF) during the severe UCO series versus 0.09 ± 0.07 (ECOG-FHR CCF) and 0.31 ± 0.14 (EEG-FHR CCF) for all previous time periods (*P* < 0.01 and *P* < 0.001, respectively, Fig.3).

To validate the synchronization (phase locking) between ECOG/EEG amplitude and FHR, we compared the coherence in the 0.01–0.1 Hz band – representing the 10–100 sec locking period – to all other frequency bands. The coherence checks the correlation removing the effects of phases. The average coherence in this band for ECOG-FHR and EEG-FHR was at 0.19 ± 0.02 versus 0.15 ± 0.04 (*P* < 0.01) and 0.19 ± 0.04 versus 0.15 ± 0.05 (*P* < 0.05) indeed significantly higher during the severe UCO series than during all previous time periods.

## Discussion

Our findings provide the physiological proof-of-principle for fetal EEG monitoring during human labor.

### Physiological considerations

Fetal responses in blood gas, acid base, and metabolic parameters observed here are similar to those reported (Itskovitz et al. 1983; Frasch et al. 2009) and have been presented elsewhere for the present animal cohort (Ross et al. 2013; Xu et al. 2015). Briefly, the mild-partial, moderate-partial, and severe-complete UCOs resulted in transitory fetal hypoxemia and hypercapnia of increasing severity due to the greater reduction in umbilical blood flow from mild to severe UCO series. All fetuses developed worsening respiratory and metabolic acidemia. Lactate levels increased 6.7-fold from 1.5 mmol/L at baseline to 9.8 mmol/L at pH nadir contributing to metabolic acidosis in the fetus (Low 1988). Each UCO is known to contribute to lactate accumulation (Richardson et al. 1996; Frasch et al. 2009). Glucose levels increased 1.7-fold from 1.2 mmol/L at baseline to 2.0 mmol/L at pH nadir. This is likely due to an increase in fetal glycogenolysis in the ovine fetus with sustained hypoxemia and a rise in catecholamine levels (Gu et al. 1985).

Noteworthy, we observed a pronounced decrease in arterial oxygenation as evidenced by the O_2_Sat, but not arterial pO_2_, with levels dropping twofold over the course of the occlusions. This is likely due to the acidemia-triggered shift in the oxyhemoglobin dissociation curve that will impact O_2_Sat, but not pO_2_. Both the falling O_2_Sat and pH levels correlated to the individual timing of ECOG/EEG-FHR pattern onset. This raises two clinically relevant questions requiring further study: (1) Why do seemingly healthier fetuses present earlier adaptive brain shut-down? (2) Since in a clinical scenario the pH nadir remains unknown, can we identify additional properties within fetal EEG or FHR that would indicate not only the incipient acidemia *per se*, but also the position of the individual fetus on the trajectory to developing acidemia, that is, can we estimate the time remaining until an individual fetus would reach pH nadir of 7.00 based on the current EEG or FHR data provided the labor is allowed to continue? We will attempt to address these questions in the following paragraph.

Fetal cardiovascular responses to repetitive UCOs followed an expected pattern: rising ΔFHR was accompanied by rising ΔABP and ΔABP_UCO_, all three parameters roughly doubling their values during severe compared to mild UCO series (Frasch et al. 2009). Interestingly, highly correlated with the onset of the ECOG/EEG-FHR synchronization, ΔABP diminished drastically with each UCO-induced FHR deceleration due to the onset of pathologic hypotension toward the end of each UCO. ΔABP reflects global cardiovascular behavior during and between the occlusions and hence captured well the drastic ABP changes, while ΔABP_UCO_ is an UCO-specific measure and did not follow the ECOG/EEG-FHR synchronization pattern. This observation validates our initial report in a different prospective fetal sheep cohort, also modeling human labor using a UCO approach, but with a slightly different paradigm: complete UCOs of increasing frequency were used rather than fixed frequency UCOs of increasing severity, as done in this study (Frasch et al. 2011). In light of the two questions posed above, we propose that cardiovascular decompensation may provide the substrate for estimating a fetus-specific time until pH nadir is reached. The observed pathologic hypotension may result from diminishing myocardial reserves under conditions of intermittent hypoxia and worsening acidemia, failure to maintain peripheral vasoconstriction, adaptive neural reflexes such as Bezold-Jarisch or a combination of these mechanisms (Hokegard et al. 1981; Rosen et al. 1986; Block et al. 1990; Giussani et al. 1993; Nuyt et al. 2001; Gardner et al. 2002b). It is possible that the earlier adaptive brain shut-down observed in healthier fetuses (with respect to arterial oxygenation and degree of acidemia) reflects their general superior capacity to redistribute blood flow under conditions of UCOs than in previously hypoxic fetuses (Block et al. 1990; Giussani et al. 1993). Moreover, hypoxic fetuses deteriorate faster than normoxic animals during UCO, as we reported (Xu et al. 2015). Hence, with the blood gas sampling every 20 min these animals had “more time” to deteriorate between the 20 min samples and therefore their most proximal sample to EEG/ECOG alarm had a lower value.

The precise mechanisms triggering the adaptive brain shut-down remain to be elucidated. Regardless the mechanisms, parameters reflecting cardiovascular decompensation and neural control, such FHR variability, may be the candidates for improving the current approach for individualized early detection of incipient acidemia.

With our previous UCO paradigm of complete UCOs with decreasing recovery time, we detected a pronounced increase of the dominant frequencies of ECOG (i.e., the 95% SEF) from the *δ*-band between the UCOs up to the *β*-band during the UCOs when the ECOG-FHR synchronization pattern emerged (Frasch et al. 2011). In this study, the SEF ECOG increase continued to be apparent in the ECOG signal, but not in the EEG signal. The SEF ECOG increase was confined to a jump from *δ*-band to *α*-band, rather than higher ECOG frequencies seen in the previous UCO paradigm. This observation suggests that mode of UCO resulted in a different pattern of adaptive neuronal shutdown with respect to the frequency characteristics, but not with respect to the amplitude characteristics. Frequency characteristics represent various neuronal populations affected by the insult, whereas the amplitude properties depend on global neuronal activation dynamics that are more likely to conceal the insult-specific differences in neuronal networks types affected. Moreover, the subtler information encoded in frequency characteristics is more likely to be “scrambled” by the artifact-prone EEG recordings compared to the ECOG signal. That would explain the lack of SEF EEG change with emergence of the EEG-FHR synchronization pattern. Overall, the frequency of UCOs does not impact on the pattern of the ECOG/EEG amplitude response that signals incipient acidemia, but it does impact the ECOG/EEG frequency characteristics.

### Translational considerations: Fetal EEG during labor may permit early detection of acidemia

Our findings confirm the notion that ECOG activity acquired from supradural electrodes and EEG activity acquired from scalp electrodes should similarly reflect the field potential neuronal activity, albeit the ECOG amplitude is larger than the corresponding EEG signals. This result complements a recent study showing the technological feasibility of online EEG-FHR monitoring and pattern detection during UCO with or without preceding hypoxia or pathological inflammation (Wang et al. 2014).

Clinical studies with cerebral function monitors in newborns with suspected hypoxic-ischemic encephalopathy have demonstrated the feasibility of recording EEG activity from scalp electrodes and predictive value for longer term neurologic sequellae (Thordstein et al. 2004; de Vries and Hellstrom-Westas 2005). However, this predictive ability relates to existent and evolving brain injury associated with necrotic/apoptotic cell death (Williams et al. 1991) and the secondary impact on EEG activity, rather than an adaptive suppression of synaptic activity as a protective mechanism. EEG activity as a measure of brain function has also been assessed in the human fetus during labor-related events after rupture of the membranes. Rosen et al. pioneered the human fetal EEG field in the 1970s using ‘suction-cup’ EEG electrodes placed transvaginally on the fetal scalp and were able to acquire brain activity during uterine contractions, epidural anesthesia and drug administration (Borgstedt et al. 1975; Chik et al. 1976; Sokol et al. 1977; Frasch et al. 2011). However, these studies were hampered by the lack of advanced computer-based technology for analyzing large data sets and the need for multiple scalp electrodes which made large-scale clinical usage impractical. Recently, Thaler et al. (2000) used real-time power spectral analysis of fetal EEG during labor to facilitate signal processing and interpretation. However, while clearly demonstrating the presence of sleep state cycles in the human fetus, this study was limited to 14 healthy pregnancies with normal outcomes, and again used multiple scalp electrodes to acquire the EEG, which is not feasible for large-scale clinical use. As such, there has been continued need to develop a single transvaginal probe capable of acquiring EEG and FHR signals as an essential first step to ensure the clinical feasibility of monitoring both for the assessment of fetal health during labor.

Our findings overcome the limitations discussed above by demonstrating in an animal model of human labor that (1) an EEG probe can be used practically and by every obstetrician trained in placing the spiral FHR scalp probe during labor; and (2) EEG-FHR monitoring is capable of early detection of worsening hypoxic-acidemia, allowing for timely intervention. As an ancillary tool for intrapartum FHR monitoring, fetal EEG monitoring should provide additional decision-making power to the obstetrician faced with question of whether to allow a labor to proceed or to deliver acutely. That discrimination ability ought to minimize the number of babies born with severe acidemia and increased risk for brain injury on one hand and decrease the number of unnecessary cesarean sections at the other hand.

Our technology is now ready to be tested in prospective clinical studies of electronic fetal monitoring during labor.

### Significance and future directions

The utility of joint EEG-FHR monitoring is based on consistent emergence of synchronized UCO-triggered EEG-FHR changes prior to reaching a severe degree of fetal acidemia at which brain injury might occur. These changes are likely due to the mechanism of adaptive brain shut-down triggered at pH of around 7.20. Noteworthy, Yumoto et al. (2005) also reported the pH value of 7.20 to be a critical value below which fetal myocardial contractility decreases. Adaptive brain shutdown prevents the brain from passing from upper to lower ischemic flow threshold (Astrup 1982; Attwell and Laughlin 2001). Past the lower ischemic flow threshold, permanent neurological injury ensues (Frasch et al. 2011). Our findings suggest that the considerable spread in the individual timing onset of the adaptive brain shut-down may at least partially be explained by the oxygenation levels at the time of the ECOG/EEG-FHR pattern onset: the more hypoxic the fetuses were at the time, the closer to pH nadir would they show the pattern. This finding may reflect an individual degree of cardiovascular adaptation during UCO: prolonged onset of hypoxia results in a prolonged onset of adaptive brain shut-down. Future studies will have to seek individual signatures of such cardiovascular adaptation contained in noninvasively obtainable signals such as FHR. Another direction of study should be to elucidate the impact of acidemia compounded by chronic hypoxia as may occur with placental insufficiency or by prolonged UCO with bradycardia on the emergence of the ECOG/EEG-FHR pattern and its utility to detect early adaptive brain shut-down in such at-risk fetuses.

We conclude that fetal EEG monitoring during labor has potential as a valuable ancillary technique of electronic monitoring, possibly warning of an incipient cardiovascular and cerebral decompensation due to repetitive UCO. Together with FHR, EEG can provide an early, inexpensive and easily implementable and interpretable tool to accurately predict incipient acidemia in fetuses.
